# Method for Handling Massive IoT Traffic in 5G Networks

**DOI:** 10.3390/s18113966

**Published:** 2018-11-15

**Authors:** Safdar Nawaz Khan Marwat, Yasir Mehmood, Ahmad Khan, Salman Ahmed, Abdul Hafeez, Tariq Kamal, Aftab Khan

**Affiliations:** 1 Department of Computer Systems Engineering, University of Engineering and Technology Peshawar, Peshawar 25120, Pakistan; ahmadkhan@uetpeshawar.edu.pk (A.K.); sahmed@uetpeshawar.edu.pk (S.A.); tkamal@uetpeshawar.edu.pk (T.K.); aftab.khan@uetpeshawar.edu.pk (A.K.); 2 Communication Networks, University of Bremen, 28359 Bremen, Germany; ym@comnets.uni-bremen.de; 3 Department of Computer Science, University of Engineering and Technology Peshawar (Jalozai Campus), Nowshera 24240, Pakistan; abdul.hafeez@uetpeshawar.edu.pk

**Keywords:** 5G, IoT, resource block, r-stage Coxian process, analytical model

## Abstract

The ever-growing Internet of Things (IoT) data traffic is one of the primary research focuses of future mobile networks. 3rd Generation Partnership Project (3GPP) standards like Long Term Evolution-Advanced (LTE-A) have been designed for broadband services. However, IoT devices are mainly based on narrowband applications. Standards like LTE-A might not provide efficient spectrum utilization when serving IoT applications. The aggregation of IoT data at an intermediate node before transmission can answer the issues of spectral efficiency. The objective of this work is to utilize the low cost 3GPP fixed, inband, layer-3 Relay Node (RN) for integrating IoT traffic into 5G network by multiplexing data packets at the RN before transmission to the Base Station (BS) in the form of large multiplexed packets. Frequency resource blocks can be shared among several devices with this method. An analytical model for this scheme, developed as an r-stage Coxian process, determines the radio resource utilization and system gain achieved. The model is validated by comparing the obtained results with simulation results.

## 1. Introduction

Internet of Things (IoT) communication has gained significant popularity in recent years. Existing IoT communication is reliant on present-day mobile networks. The current networks are meeting the requirements of existing IoT devices sufficiently to date. However, the massive growth of IoT data traffic means that the existing mobile communication systems (3G, 4G, etc.) might not be capable of coping with substantial future data traffic. IoT traffic is usually dissimilar to regular traffic such as conversational video or file transfer. IoT data are mostly generated by a large number of IoT devices in the form of small packets. IoT communication is mostly based on narrowband applications. Conversely, the human-generated normal data traffic appears from small number of mobile phones in the shape of large sized packets. Meanwhile, 5G is now being considered as a strong candidate for serving the massive future IoT traffic. Rollouts of 5G networks are expected in the near future.

The 3rd Generation Partnership Project (3GPP) standard, Long Term Evolution-Advanced (LTE-A) has been developed for broadband applications. With narrowband applications, LTE-A is not capable of achieving efficiency in terms of bandwidth usage and cost. Incorporation of the narrowband IoT data traffic into LTE-A system requires revision in the design of such networks. Otherwise, considerable degradation of the overall network performance can be faced. Therefore, achieving high spectral efficiency in air interface requires intelligent use of scarce radio resources. Regular human-based broadband traffic in LTE-A can efficiently utilize radio resources with transmission of large messages via a single resource block of frequency. However, these resource blocks may be underutilized while serving narrowband IoT applications due to the transmission of small-sized messages with transmission gaps. As per 3GPP specifications, each device must use at least one resource block for transmission of a message without sharing the block with other devices.

Instead of allowing IoT devices to access the Base Station (BS) directly, a Relay Node (RN) could be used to accumulate data packets transmitted by IoT devices. RN is deployable without adding infrastructure for backhaul connection and can be connected wirelessly to the BS. This work investigates a framework for aggregating IoT data from a group of IoT devices and then multiplexing the traffic at RN before sending to the BS. Thus, rather than demanding frequency resources from the BS for singular IoT devices, resource requests are executed by RN for groups of IoT devices collectively. BS would consider a resource demand for aggregated data as a solitary request. The effectiveness of the method is established with the help of an analytical model.

## 2. Literature Survey

The literature available on RN indicates that research work in this field is mostly focused on implementation aspects and improving system performance [1,2,3,4]. Performance of relaying in mobile networks has been studied by several researchers. In [5], algorithms for energy efficiency enhancement and optimum node placement are proposed. In [6], challenges related to mobile communication in high-speed trains such as attenuation and high Doppler spread are addressed. In [7], power control mechanisms are investigated for enhancing the outage performance of relaying scheme via centralized and distributed power adjustment schemes. Performance evaluation of relaying systems for the end-to-end delay is studied with the help of semi-Markov processes in [8]. The derivation of analytical results for the expected packet end-to-end delay is also explored in [8]. An investigation of connectivity and service interruption time before reconnecting to RN in 4G networks for handhelds and relays is carried out in [9]. Data security issues during handover in mobile networks are addressed in [10] using mobile relaying. Deployment of an unmanned aerial vehicle as a relaying node in wireless communication is explored in [11].

Research work available on packet multiplexing is mostly based on 4G and previous mobile communication systems. Statistical multiplexer’s performance for the determination of packet delay arising in voice traffic is studied in [12] using Markov modulated Poisson process. The authors of [13] discuss an end-to-end packet delay performance of data encapsulation as well as packet aggregation technique with arrivals having Poisson distribution and service time with phase-type distribution. As of multiplexed flows, approximation methods for finding probability distribution function for packet waiting time are presented in [14]. In [15], the authors present a scheme for packet aggregation to handle M2M data traffic having small-sized messages by sharing radio resources of LTE-A in downlink; however, uplink M2M traffic is not considered. In [16], an analysis of aggregation delay for packets from multiple sensors with on-off type of data traffic in Wireless Body Area Networks is presented. In [17], the authors present a framework for dimensioning of the 5G access network by utilizing G/G/1 queuing models for delay percentiles. In [18], capacity development for packet-based radio access technologies through multiplexing gain is investigated. The authors conclude that multiplexing gain is achievable for traffic with variable bit rate but not for traffic with constant bit rate. A Software Defined Network (SDN)-based approach to packet aggregation and then disaggregation after transmission is presented in [19].

The Asynchronous Transfer Mode (ATM) Adaptation Layer 2 (AAL2) represents a technique for multiplexing of packets in networks such as 3G. In this technique, several small packets are fitted into large ATM cells. In [20], the authors present a probabilistic model of AAL2 for the 3G core network. In [21], a queuing model for the evaluation of AAL2 packet multiplexer performance modelled as batch Markovian arrivals having timer mechanism is presented. Extension of [21] presented in [22] is for modelling of multiplexing buffer. Authors of [23] present a mathematical model for evaluation of AAL2 type multiplexer’s performance parameters like expected sojourn time, mean queue length, and the probability of delay violation. Authors of [24] analyze the AAL2 multiplexer by modelling the multiplexer departure process as an r-stage Coxian process. The authors of [25] present the performance evaluation of voice traffic in mobile radio networks with Coxian distributed channel holding time. The authors of [26] propose a method of performance analysis for a continuous time Markovian arrival process at the ATM Adaptation Layer 2 (AAL2), where the Common Part Sublayer (CPS) packets get multiplexed at the AAL2 buffer before being sent to transmission buffer. Both the buffers are modelled as queues.

## 3. Air Interface of 5G

The primary motivation behind the development of new standards for 5G mobile systems is innovations in support of IoT traffic. 5G applications for IoT are expected to increase market space and open new profitable avenues for operators. Furthermore, improvement in customer experience would also generate sizeable returns. The requirements laid out for 5G performance include enhancing the capacity by 1000 times, data rate by 100 times, a latency of less than a millisecond and 1000 times lower energy consumption. These requirements imply that significant challenges in the design of network architecture and air interface lie ahead.

**Figure 1:**
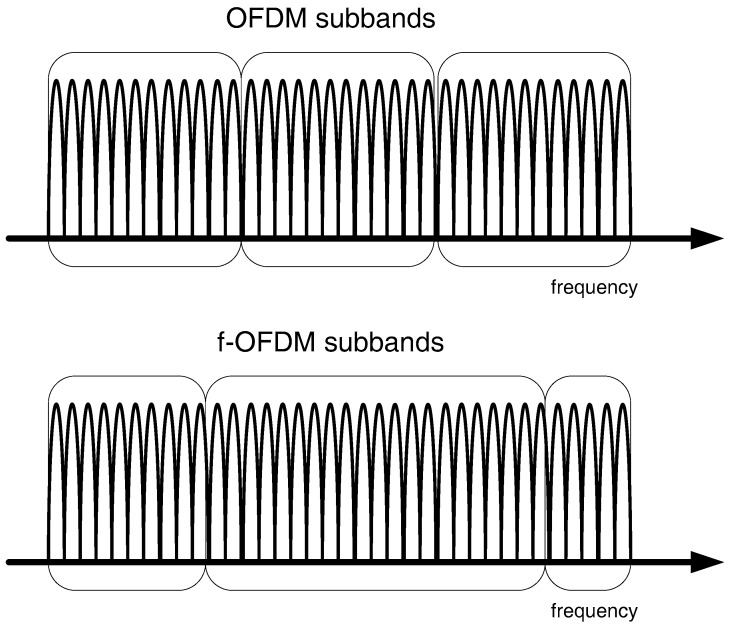
OFDM vs. f-OFDM subband sizes.

In mobile communications, air interface is the crux of all the novelty undertaken in the development of a system. The progression in designing air interface schemes for 5G systems has taken considerable time over the years. Currently, filtered-OFDM (f-OFDM), which facilitates flexible waveform configuration, has been tipped as the 5G air interface scheme [27]. Under normal OFDM as in LTE-A, a specific numerology is used for the whole bandwidth, which results in limited spectral efficiency [28]. Under f-OFDM, the bandwidth is divided into several subbands for various types of services with appropriate numerology, resulting in enhanced utilization of spectrum (Figure 1).

## 4. Relay Node Design and Scheduling Scheme

The RN has been introduced by 3GPP in Release 10 documentation [29]. RN is designed for extending the cell coverage area. The RN appears as a low-power BS to the mobile device. The RN is wirelessly linked to devices and the BS. The cost of deploying RN is less than deploying femto- and picocells, as no additional infrastructure is needed for connecting RN to the BS. RN improves coverage when placed at positions with weak channel conditions or dead spots in the coverage area.

In this work, a wireless, fixed, inband, layer 3 type RN is proposed as the data aggregation and multiplexing node. This type of RN can be designed to aggregate and multiplex data packets from a large number of IoT devices. The proposed inband RN operates on the same radio frequency band that is specified to the BS by the network service provider. For accomplishing effective incorporation of IoT traffic in the 5G system, functionality of RN is modified slightly in this work.

Additionally, an RN Medium Access Control (MAC) packet scheduler [30] is used in this work to allocate frequency resources to IoT devices for RN access. The scheduler is capable of allocating resources to devices with various Quality-of-Service (QoS) traffic classes. In this work, the scheduling scheme used is blind equal throughput. This scheduler works in addition to the MAC layer scheduler placed at the BS for allocating resources to mobile devices and RNs connected to BS. It is also assumed in this work that all the IoT devices are having similar QoS requirements, therefore service differentiation is not performed. The BS scheduler in [31] is also employed in this work at the BS.

**Figure 2:**
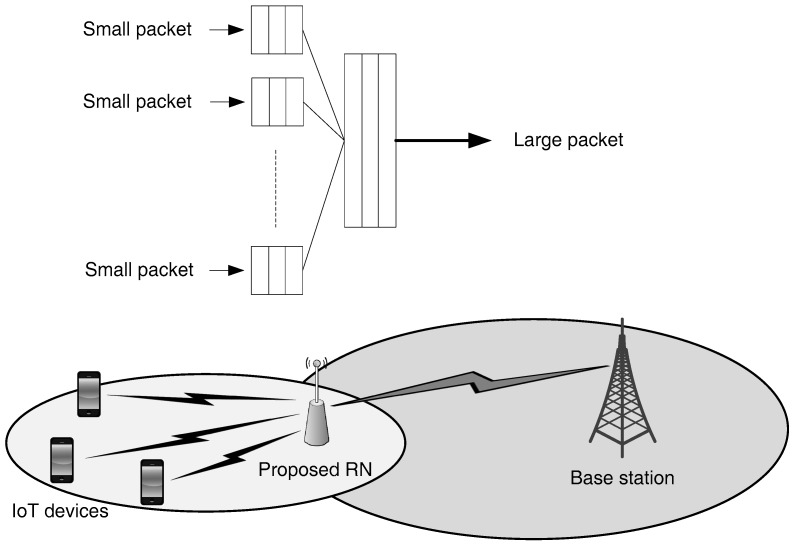
RN-based data multiplexing scheme.

A multiplexing algorithm at RN is developed for aggregation of IoT traffic from different devices where packets are multiplexed before transmission to the BS. The multiplexing algorithm works collaboratively with the scheduler at RN to ensure that the size of multiplexed data is in accordance with the capacity of available radio resources (Figure 2).

Thus, rather than demanding frequency resources from the BS for distinct IoT devices, resources are demanded by the RN for a bunch of IoT nodes. The IoT packets are aggregated and multiplexed at the RN. This multiplexing is achieved by, firstly, aggregating packets from several IoT devices and then multiplexing them into one large packet. On the BS side, a resource demand for the multiplexed large packet would be considered as one distinct radio resource demand. Therefore, sharing of radio resources among several devices would result in the enhancement of spectral efficiency. The capacity of the network would also increase, resulting in minimization of chances of network failure.

## 5. Data Aggregation and Scheme for Multiplexing

In this work, the design of a fixed RN is presented for 5G networks. IoT data traffic from devices located near RN is aggregated at the RN. In this paper, it is supposed that the RN antennas for the access link (interface with devices) and the backhaul link (interface with BS) are well separated and that self-interference can be totally avoided. For example, consider the case of deploying the access antenna inside some building while the backhaul antenna is deployed outdoors. In such cases, the inband operation of RN can be ensured without any time division mechanism. At the MAC layer of the BS, a channel and service aware scheduler [31] is implemented for scheduling the RN as well as mobile user devices with regular data traffic. The scheduler consists of time and frequency domain uplink scheduling schemes.

**Figure 3:**
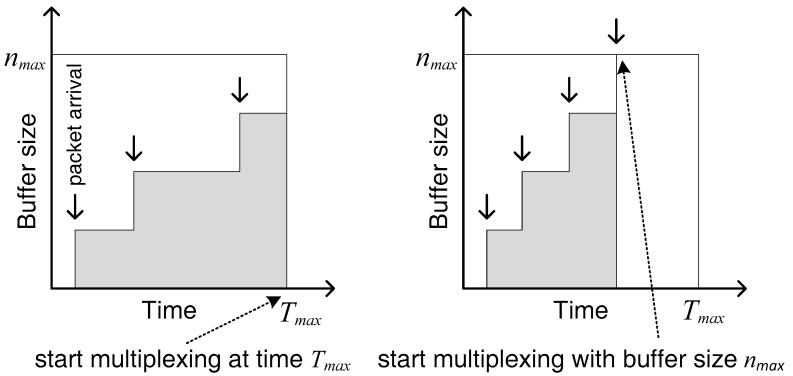
Multiplexing either due to timer expiry or maximum buffer size.

An aggregation buffer is used at the RN for aggregating IoT data having a size that matches the size of instantaneously achievable Transport Block Size (TBS) offered by the air interface. The maximum buffer size denoted by $n_{max}$ corresponds to instantaneously available TBS, which depends on channel conditions. However, the multiplexed data forming a large packet can have a maximum size of $n_{max}-overhead$ (from lower layers). The overhead of LTE-A lower layers is assumed to be 352 bits [32]. The multiplexed large packet is transmitted to the BS via the backhaul link. This approach can bring significant improvement in the radio resource utilization, as discussed later. Conversely, this approach also can introduce problems due to constraints related to latency requirements associated with high priority data traffic, for example the delay sensitive emergency warnings. The small packets from IoT devices must wait until the buffer size reaches $n_{max}-overhead$. In case of a highly loaded traffic scenarios, this issue may not occur, as the arrival rate is high and filling the buffer with packets would not take long. The issue arises in scenarios having low traffic load, where large interarrival times can cause longer waiting times greater delays in stuffing the buffer. As a result, the performance of delay sensitive IoT applications is compromised. This issue is dealt with by deploying a timer in the scheme. The timer is set to a certain time $T_{max}$, which is the maximum waiting time of the first packet arriving since the empty buffer (Figure 3). Thus, the buffer multiplexes the aggregated packets until a duration $T_{max}$ of the first arrival. Once waiting time of a packet reaches the timer expiry duration $T_{max}$, the process of packet multiplexing is triggered, and the large multiplexed packet is sent to the BS. The original small packets from the IoT devices are then sent from the BS to the core network.

## 6. Analytical Model

The small IoT data packets from IoT devices arrive at the RN multiplexer. The first packet arrival at the multiplexer activates the timer. Due to the timer activation, the first packet would stay in the buffer for a duration not more than $T_{max}$. During this time, arrival of additional packets would result in improvement of multiplexing gain. Once the packet waiting duration reaches the timer expiration threshold $T_{max}$, the course of packet multiplexing is triggered. The large multiplexed packet is then transmitted to the BS. $N_{max}$ is the maximum number of resource blocks available for RN to use in a single Transmission Time Interval (TTI). If some or all of the $N_{max}$ radio resource block are not needed in a TTI for RN traffic, the unallocated blocks are made available for regular mobile users waiting for radio resources from the BS. Alternately, if the timer expiry time $T_{max}$ has not been reached, but the total size of small packets arrived at the multiplexing buffer reaches the size of $n_{max}-overhead$; the large multiplexed packet is formed then and sent to BS before the timer expires. The size of $n_{max}$ depends on the available TBS, which in return is dependent on backhaul air interface radio channel conditions. At most, r packets can be aggregated into a single large packet and r is expressed as:

(1) $$r=\frac{n_{max}-overhead}{l}$$

where l describes the fixed size of the arriving packets in bits. Consider x as the actual number of packets in the RN buffer. As long as the waiting time does not reach $T_{max}$ or the buffer size does not reach $n_{max}-overhead$, x remains in the interval 1≤x<r. Packet arrival rate from IoT devices, λ is considered to be exponentially distributed. It can be shown that the total waiting time of $x^{th}$ packet is Erlangian [24]. Therefore, the probability of subsequent arrival of packet prior to multiplexing with x packets already in the buffer is denoted as $a_{x}$ and given as:

(2) $$a_{x}=\{\begin{array}{cc} 1, & x=0\\ 1-\left(\overset{x-1}{\underset{i=0}{\sum }}\frac{e^{-\lambda T_{max}}{\left(\lambda T_{max}\right)}^{i}}{i!}\right), & 1\leq x<r\\ 0, & x\geq r \end{array}$$

**Figure 4:**
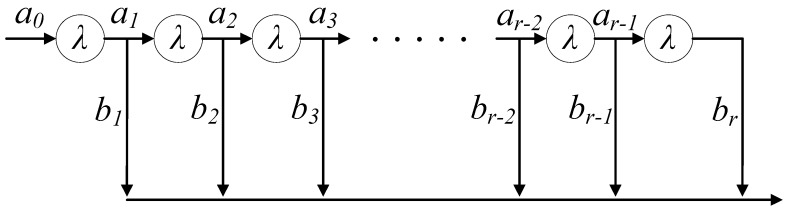
r-stage Coxian process.

The probability of initiating the multiplexing after x arrivals is $b_{x}$ where $b_{x}=1-a_{x}$. This multiplexer now emulates an r-stage Coxian process (Figure 4). The probability of an arrival with empty buffer, $a_{0}$ is 1 as the packet multiplexing starting without a single packet in the buffer is not possible. For triggering the multiplexing process, a single packet must arrive at the multiplexing buffer. Consequently, $b_{0}$ would always be 0. Upon arrival of the first packet from IoT device, the multiplexing system goes to stage 1 of the Coxian process with a total of r stages. Stage 1 illustrates that one packet has arrived in the buffer. During the first stage, two cases are possible. It is likely that the second packet also arrives into the multiplexing buffer ahead of timer expiry. The probability of this case is denoted as $a_{1}$. But the converse case can also happen where no packet arrivals occur after the first one until timer expiry and the packet multiplexing is initiated with a single packet in the buffer. The probability of this case is expressed as $b_{1}$. Now, if the second packet arrival occurs before the timer expires, the process is said to have entered stage 2. After entering stage 2, again two cases are possible. Either the third packet arrival occurs, which is considered to have a probability $a_{2}$. Or the timer expires after the second arrival with a likelihood of $b_{2}$. These probabilities $a_{x}$ and $b_{x}$ in each stage could happen only until x becomes equal to r, i.e., r packets arrive. As discussed earlier, r is the highest possible number of small packets that the buffer holds until starting the multiplexing process. Upon entering stage r, the sole probable event that can happen then is that the process of packet multiplexing begins immediately, and no more packet arrivals are awaited. At stage r, $a_{r}=0$ while $b_{r}=1$.

The packet multiplexing process modelling is particularly useful in cases of data traffic where packet arrivals occur in bursts in between relatively silent periods. The generation of traffic at IoT devices is statistically independent. Thus, the traffic arriving at RN would be like bursts followed by periods of comparative silence. The goal of multiplexing is to combine the incoming packets at RN and make large blocks of data rather than sending isolated packets. This arrangement can reduce the number of blocks sent to BS considerably. The number of resource blocks needed for each stage can be determined by checking the TBS value for different resource block allocations and Modulation and Coding Scheme (MCS) combinations. Thus, the resource blocks required for different buffer sizes at the time of multiplexing is derived from the TBS table of 3GPP. The TBS values for M=16 (as per 3GPP specifications [33]) and the buffer capacity in terms of data without overhead (352 bits) are given in Table 1 below.

At stage 1, the buffer size in bits can reach l bits, where l is the fixed size of the arriving packets in bits. In this way, at stage x, the size of buffer would become x×l bits. The number of resource blocks needed for each stage x is denoted as $N_{x,M}$, which relies on stage x and MCS M. The values of $N_{x,M}$ for M=16 and l=232 bits are given as in Table 2 below.

To evaluate the performance of the proposed mechanism analytically, a discrete Random Variable (RV) X is defined, which denotes the number of packets fitted into a single multiplexed packet. The RV X can have possible values x=1,2,3,…,r. Here, r can also be defined as the maximum number of IoT data packets inside a single multiplexed large packet. P[X=x] denotes the probability mass function of RV X. The likelihood of having more than x packets multiplexed into a large packet is $a_{x}$. So $a_{x}$ is now defined in another manner. The probability $a_{x}$ represents the chance of having a value of X greater than x. This implies that the chance of getting more than x IoT data packets inside a multiplexed large packet is $a_{x}$, which is given in the form of P[X>x] for 1≤x<r as:

(3) $$\begin{array}{c} P\left[X>x\right]=1-\left(\overset{x-1}{\underset{i=0}{\sum }}\frac{e^{-\lambda T_{max}}}{i!}{\left(\lambda T_{max}\right)}^{i}\right)\\ for\;1\leq x<r \end{array}$$

This is illustrated with an example. Consider that $a_{1}=0.6$, which implies that 60% of the multiplexed large packets should be having a size greater than one small packet, which indicates that P[X>1]=0.6. Now the likelihood of X>0 with x having value of 0 must always be 1, i.e., P[X>0]=1. This is due to the fact that multiplexing requires a minimum of one packet in the multiplexing buffer, which means that the value of X must be above 0 if multiplexing has to occur. Now by setting x=0 in Equation (3), P[X>0] turns out to be 1. In this fashion, the chance of X greater than or equal to r, i.e., P[X≥r] is always 0.

Since, X is a RV of discrete values, the likelihood P[X=x] is established as:

(4) P[X=x]=P[X>x−1]−P[X>x]

This gives P[X=x] as:

(5) $$P\left[X=x\right]=1-\overset{x-2}{\underset{i=0}{\sum }}\frac{e^{-\lambda T_{max}}}{i!}{\left(\lambda T_{max}\right)}^{i}-\left(1-\overset{x-1}{\underset{i=0}{\sum }}\frac{e^{-\lambda T_{max}}}{i!}{\left(\lambda T_{max}\right)}^{i}\right)$$

(6) $$\Rightarrow P\left[X=x\right]=-\overset{x-2}{\underset{i=0}{\sum }}\frac{e^{-\lambda T_{max}}}{i!}{\left(\lambda T_{max}\right)}^{i}+\overset{x-1}{\underset{i=0}{\sum }}\frac{e^{-\lambda T_{max}}}{i!}{\left(\lambda T_{max}\right)}^{i}$$

(7) $$⟹P\left[X=x\right]=-\overset{x-2}{\underset{i=0}{\sum }}\frac{e^{-\lambda T_{max}}}{i!}{\left(\lambda T_{max}\right)}^{i}+\overset{x-2}{\underset{i=0}{\sum }}\frac{e^{-\lambda T_{max}}}{i!}{\left(\lambda T_{max}\right)}^{i}+\frac{e^{-\lambda T_{max}}}{\left(x-1\right)!}{\left(\lambda T_{max}\right)}^{x-1}$$

(8) $$⟹P\left[X=x\right]=\frac{e^{-\lambda T_{max}}}{\left(x-1\right)!}{\left(\lambda T_{max}\right)}^{x-1}$$

## 7. Spectrum Utilization

Using $N_{x,M}$ from Table 2 and P[X=x] from Equation (8), the average resource blocks used in each multiplexing process or the average resource blocks consumed by a large packet is denoted by k, and determined as:

(9) $$k=\overset{r}{\underset{x=1}{\sum }}N_{x,M}\times P\left[X=x\right]$$

The mean number of resource blocks consumed by a single multiplexed large packet, k is dissimilar from the parameter, mean number of blocks utilized per TTI. The mean number of resource blocks per TTI are considerably decreased by employing the multiplexing scheme along with the timer. However, k increases with usage of packet multiplexing scheme. The greater the value of k, the better the resource utilization. The multiplexing of packets happens if a duration $T_{max}$ has elapsed. If the packet aggregation scheme is not used at the RN and packets are sent to BS without multiplexing, then the arriving packets would be sent to BS immediately at the beginning of the next TTI, instead of waiting for the timer to expire. For this reason, the average number of resource blocks for each large packet would increase in case of multiplexing with the timer. As a result, the total number of multiplexed packets sent from RN to BS would be reduced considerably. The lesser the number of multiplexed packets, the higher the value of k and the lesser the overhead data.

## 8. Multiplexing Gain

Multiplexing gain is achieved by increasing the resource block utilization in every multiplexing process. For determining the multiplexing gain, the average resource block usage k without multiplexing (represented by $k_{no\_mux}$) as well as with multiplexing (represented by $k_{mux}$) are determined in accordance with Equation (9). Multiplexing gain in percentage is given as:

(10) $$G=100-\left(\frac{k_{no\_mux}}{k_{mux}}\times 100\right)$$

## 9. Simulation Model

The analytical results of the derived model for resource block utilization are compared with simulation results for resource block usage. The simulation results in this paper are generated under simulation parameters given in Table 3. The evaluation underlines the case of relaying without multiplexing and the impact of relaying with multiplexing as well as timer mechanism. The two cases are compared to determine the multiplexing gain achieved both in analytical and simulation models.

**Figure 5:**
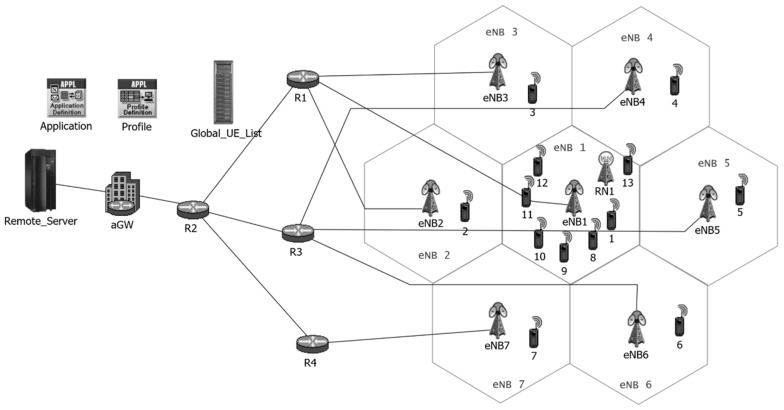
OPNET project model of hexagonal grid [35].

The OPNET simulation environment (Figure 5) is used for determination of resource block usage and multiplexing gain. The OPNET model developed in this work emulates the impact of fast fading, slow fading, path loss, and noise on transmitted signal. It also models the interference from neighboring BSs. Channel models and interference models are developed according to [34]. The frequency reuse factor is 1. Therefore, devices transmitting uplink signals over certain resource blocks would cause interference in the transmission of devices of the neighboring BSs using the same resource blocks.

European Telecommunications Standards Institute (ETSI) based path loss models for different environments are used for research purposes. ETSI vehicular test environment model has been used in this work for modelling of path loss.

(11) $$L=128.1+37.6\log_{10}\left(R\right)$$

where L is path loss in dB and R is the distance between receiver and transmitter in km.

For slow fading, the log-normal model with standard deviation of 10 dB, mean 0 and correlation of 1 over distance is used. For fast fading, a time-dependent random function known as Jakes’ model is used. Thus, maps for coverage areas (cells) are generated using models in [34] and traces of these maps are fed into our OPNET simulation model. Noise and noise figure are modelled as per 3GPP specifications. Interference is modelled with in such a way that signals arriving from neighboring cells after reduction of power due to path loss, slow fading, and fast fading cause interference.

**Figure 6:**
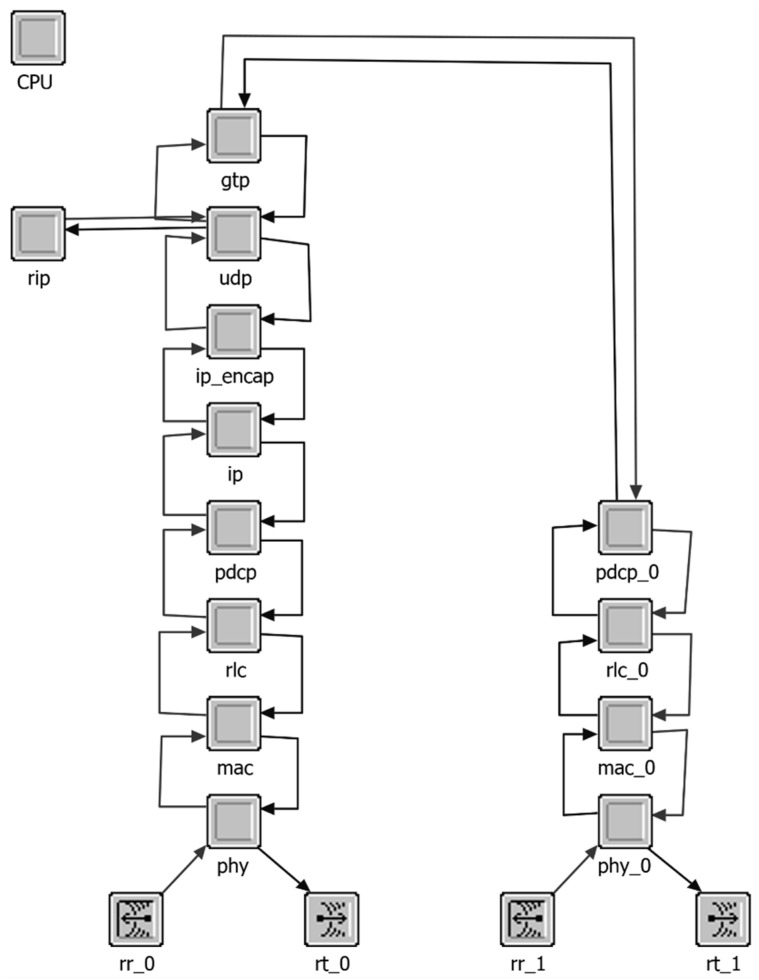
OPNET RN model.

The RN OPNET model for relaying node is implemented to feature two radio interfaces, the Uu and the Un interfaces (Figure 6). The corresponding protocol stacks are implemented as per 3GPP end-user protocols. The relaying scheduler is implemented in the MAC layer of RN Uu interface. The proposed packet aggregation and multiplexing scheme is implemented at the Packet Data Convergence Protocol (PDCP) layer of RN Uu interface. The GPRS (General Packet Radio Services) Tunneling Protocol (GTP) is also implemented where packet are tunneled at the RN and detunneling is performed at BS.

In the simulation model, the multiplexing transition probabilities are figured out by using a method of packet counting where $a_{x}$ is the likelihood of (x+1) th packet arrival prior to the start of packet multiplexing such that x packets are already present in the buffer and 1≤x<r. In the simulation model, for determining the values of $a_{x}$, r is considered as the maximum number of small data packets in the buffer multiplexed to form a large packet. So x could get a value in the range 1≤x<r.

The analytical probability, $a_{x}$ is defined in (12). To find the a-posteriori probability $a_{x}$ for simulation model, a record of the occurrences of a certain number of IoT packets packed inside a large packet is kept. For this purpose, a counter is deployed for recording arrivals at every stage of the aggregation buffer x. This means that a record of the number of instances where the Coxian process goes into a specific stage x is maintained. The counters designed for this purpose are denoted as $c_{x}$ for 1≤x<r stages and defined in such a way that $c_{x}$ counts the number of times the large aggregated and multiplexed packet contained x packets or more. Thus $c_{x}$ is also the frequency instances that a particular stage x has been reached by the Coxian process.

Probability of $a_{x}$ is found with the help of value at counter $c_{x}$. For example, to determine the probability $a_{1}$, the value at counter $c_{2}$ is considered. This figures out the likelihood of arrival of second packet with respect to value at counter $c_{1}$ (i.e., the total number of arrivals). So, $a_{x}$ is calculated as:

(12) $$a_{x}=\{\begin{array}{cc} 1, & x=0\\ \frac{c_{x+1}}{c_{x}}, & 1\leq x<r\\ 0, & x\geq r \end{array}$$

## 10. Results and Analysis

In terms of $k_{no\_mux}$ and $k_{mux}$, comparison of analytical and simulation results for various traffic load scenarios are performed. The evaluation highlights the case of relaying without multiplexing and the impact of relaying with multiplexing plus timer mechanism. The performance of the multiplexing scheme is evaluated for scenarios with 100, 200, …, 900 up to 1000 IoT devices connected to the RN. These scenarios offer traffic load of less than 0.1. The reason for evaluating performance only in low load scenarios is that the expiry of timer rarely occurs in high load scenarios. The multiplexing process would almost always start before the timer expiry. The maximum load in the simulated scenarios is 0.07 for 1000 IoT devices in the RN coverage area.

**Figure 7:**
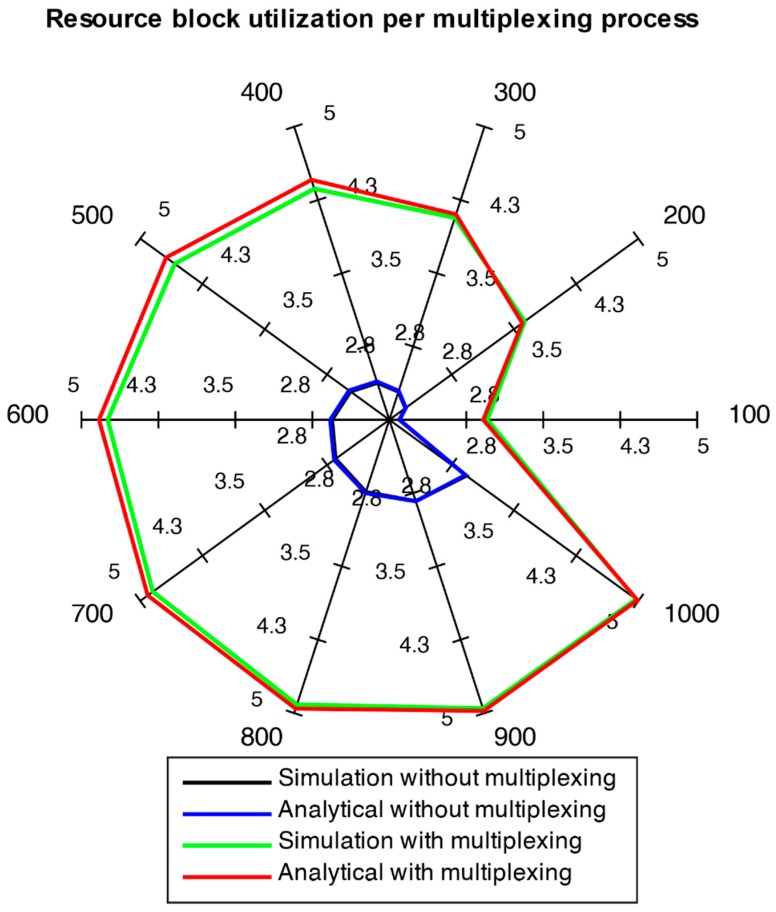
Analytical and simulation comparison of k with/without multiplexing (blue curve overrides black curve).

**Figure 8:**
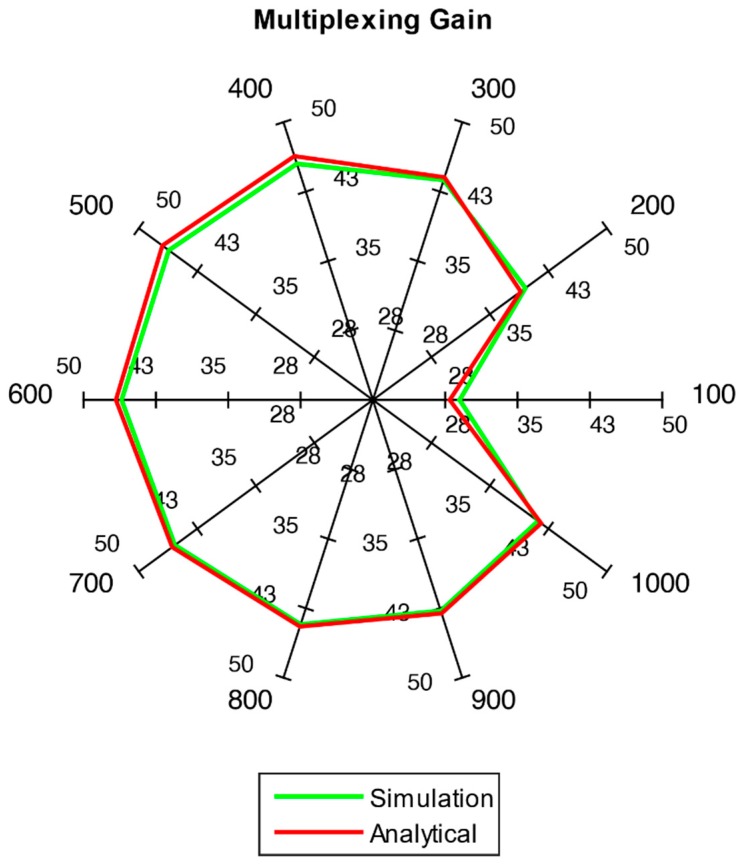
Comparison of multiplexing gain in analytical and simulation models10. Relaying Delay.

Results are depicted using spider web charts. Such a chart is used to illustrate outcomes of multiple load scenarios in a single figure. Each chart axis represents one of 10 scenarios (i.e., 100, 200, …, 1000 users). Each axis signifies a scale of 1 to 5 (Figure 7) for resource block utilization and 1 to 50 (Figure 8) for multiplexing gain percentage.

Figure 7 illustrates two aspects. First, the comparison of results for relaying with as well as without multiplexing depicts that the utilization of frequency resources is considerably improved. In the no multiplexing case, the average resource usage k is low. This shows that the total of multiplexed packets sent to BS from RN would be quite large. Therefore, the overhead data would grow significantly. The results for multiplexing with timer scenarios show that since packets are multiplexed into larger blocks, overheads needed for transmission blocks are significantly reduced. The second aspect is that the analytical results and simulation results agree with each other quite well. This conformity between analytical and simulation results indicate the validity of models. Simulation models are developed on the basis of artificial Random Number Generators (RNGs) for generation of random sequences of random values. Hence, simulation runs with various seeds for RNGs are performed to get results from varying random sequences. The 95% confidence intervals for simulation results are provided here in Table 4.

The achieved multiplexing gain G for all the traffic load scenarios is shown in Figure 8. Significant gains are achieved in all scenarios. Thus, it can be concluded that the framework provides better spectral utilization when IoT data traffic is sent over the network. It can also be noticed that the results for both simulation and analytical model agree with each other in Figure 8.

The M2M data traffic aggregation and multiplexing scheme introduces relaying delay to the uplink data packets. The evaluation of relaying delay is achieved by considering 3GPP MCS index 26, which implies relatively good channel conditions. The simulations are performed for two categories of environment settings. In one category, the simulations are performed without relaying and in the second category, simulations runs are carried out with RN for aggregation and multiplexing. The simulation parameters in addition to Table 3 parameters are provided here in Table 5. The simulations are performed for 15 scenarios, where the traffic load in each scenario varies from 1000 IoT devices up to 15,000 IoT devices. In the first scenario, the number of M2M devices is 1000. In each successive scenario, the IoT devices are increased by 1000.

The load of data traffic, which is the ratio of arrival rate and service rate, is given in Table 6 for each scenario with various number of M2M devices.

**Figure 9:**
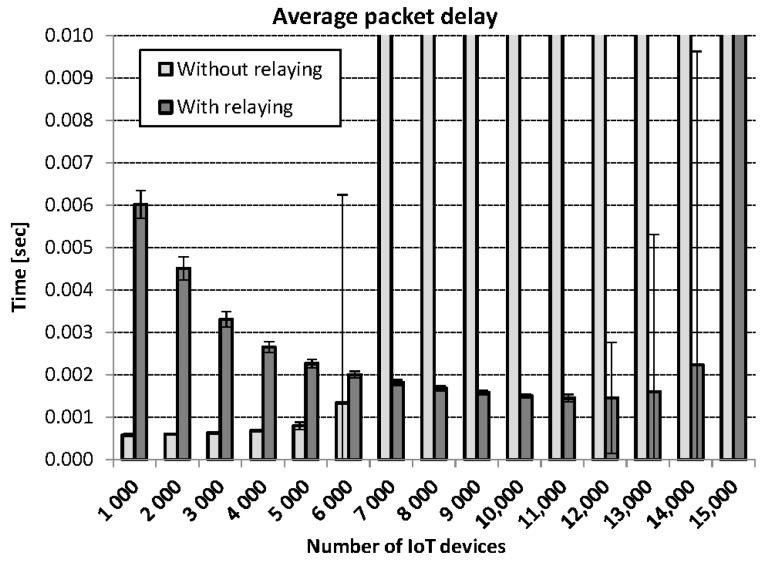
Average IoT packet delay without and with RN.

The packet delay performance of the system in both categories is compared in Figure 9. Error bars in the figure depict standard deviation. In low load scenarios, the delay performance without relaying is better than with relaying. The logic behind this behavior is that RN has to wait until arrival of $n_{max}-overhead$ bytes at RN, while no such waiting time is required if there is no relaying. However, once the traffic load increases, the environment without relaying fails to handle the load and large average delays (that do not fit to the scale used in Figure 9) are observed. In the scenario of 7000 IoT devices, this notion is clearly illustrated. However, in the environment with relaying, the traffic load of even up to 14,000 IoT devices is efficiently handled.

## 11. Conclusions

This paper provided a method for employing a low-cost 3GPP standardized layer-3, inband, RN for integration of IoT data traffic into 5G network. An RN design for aggregating packets and multiplexing IoT data before sending to BS was investigated in this work. An analytical model for data aggregation and multiplexing scheme was presented to determine resource block usage and multiplexing gain mathematically. The objective of the simulation model was justification of results achieved via analytical model using the arithmetic method. Results from both analytical and simulation models agreed with each other. Additionally, the simulation results for relaying delay performance were also illustrated. The analytical model proposed in this work provided a base for further development of analytical model for determination of transmission delays. The system performance evaluation with diverse traffic of various classes can also be derived in future.

## Figures and Tables

**Table 1:** TBS capacity for resource block values with M=16.

Number of Resource Blocks	1	2	3	4	5
TBS (bits)	328	632	968	1288	1608
Capacity without overhead (bits)	0	280	616	936	1256

**Table 2:** Resource blocks required for stages with M=16 and l=232 bits.

Stage Number	1	2	3	4	5	6
Size of buffer (bits)	232	464	696	928	1160	1392
Resource blocks	2	3	4	4	5	5

**Table 3:** Simulation parameters.

Parameter	Value/Setting
Cell layout	7 BSs, 1 RN connected to central BS
System bandwidth	5 MHz
Adjacent BS distance	500 m
Max IoT device power	23 dBm
Path loss	$128.1+37.6\log_{10}\left(R\right)$
Slow Fading	Log-normal shadowing, mean 0 dB, standard deviation 10 dB, correlation 1
Fast Fading	Jakes’ model
Noise per resource block	−120.447 dBm
Noise floor	9 dB
Power Control	Fractional, α = 0.6, $P_{0}$ = −58 dBm
Timer expiry $T_{max}$	9 ms
IoT message size	Constant (29) bytes including upper layers overhead
Message inter-transmission time	1 s (exponential)

**Table 4:** 95% confidence intervals for simulation results in Figure 7.

**Number of Devices**	**100**	**200**	**300**	**400**	**500**
Without multiplexing	0.0012	0.0007	0.0008	0.0012	0.0010
**Number of Devices**	**600**	**700**	**800**	**900**	**1000**
Without multiplexing	0.0017	0.00127	0.00097	0.00077	0.0011
**Number of Devices**	**100**	**200**	**300**	**400**	**500**
With multiplexing	0.0031	0.0026	0.0020	0.0015	0.0022
**Number of Devices**	**600**	**700**	**800**	**900**	**1000**
With multiplexing	0.0011	0.0008	0.0007	0.0005	0.0003

**Table 5:** Simulation parameters.

Parameter	Value/Setting
MCS	26
Maximum RN resource blocks	5
Number of IoT devices	1000, 2000, 3000, …, 15,000

**Table 6:** Simulation parameters.

IoT Devices	Traffic Load
1000	0.07
2000	0.14
3000	0.2
4000	0.27
5000	0.34
6000	0.41
7000	0.47
8000	0.54
9000	0.61
10,000	0.68
11,000	0.74
12,000	0.81
13,000	0.88
14,000	0.95
15,000	1.01
